# Strict Product Liability of Medical Employees for Damage Caused by the Use of Medical Means with Deficiency in the USA Law

**Published:** 2018-07

**Authors:** Samir MANIĆ, Sabina ZEJNELAGIĆ

**Affiliations:** Faculty of Law, State University of Novi Pazar, Novi Pazar, Republic of Serbia

## Dear Editor-in-Chief

In most legal systems, at the current level of development of legal relations, there is a tendency view for the damage caused by the deficiency of products, and it existed at the time of its selling period, objectively its producer bears the responsibility. Besides, an evident fact that medical means have product’s character, there is a question related to damage a patient suffers from a medical means with deficiency, can beside a producer, also be responsible a health employee/health institution that by using such means caused damage to a patient? It is an undeniable fact that for damage from medical means with deficiency, besides its producer, a health employee should also be responsible, whose quilt can be assigned damage, too. However, one part of legal and court practice in the USA advocates that besides a producer and health employees/institutions are to be objectively responsible for damage from medical means with deficiency (1).

During reconsidering the issue of expanding objective responsibility for damage from medical means with deficiency onto hospitals, the Courts were forced to reconsider the issue whether hospitals are characterized as sellers of products or as service providers (1)? In fact, the old system of values was abandoned that experienced hospitals as charitable institutions and Courts started to re-examine a new function of hospitals: hospitals as companies (2). On the one side, the initiated view emphasized the fact that health care today counts such things as medicines, medical means, blood, and also organ transplantation, etc. However, hospitals also provide health protection and, in correlation with powers, this fact stands on the other side. In the essence, when the responsibility of medical employees is on for damage from medical means with deficiency, a question can be asked whether in a certain health protection prevail goods or services (2, 3).

The Courts in the USA, in the beginning, established a parallel between *selling of products and service providing*, and they brought decisions depending on the essence of relations prevailing among hospitals, i.e. doctors and patients. Generally, the essence of a relation between a doctor-patient makes providing of services, with the ascertainment that a hospital’s work cannot be characterized as selling of products. Based on this, the Courts refused to expand objective responsibility for damage from medical means with deficiency onto hospitals (1, 4).

However, health profession contains in itself, both services providing and selling certain products, and it is impossible to establish a clear distinction between them. We can only talk on what prevails during a health “transaction”, whether it is selling of products or service providing; i.e. clearly speaking, the answer should be sought in the question what makes the essence of a certain health “transaction”, is it a certain product or, still, a certain service?

Starting from this view, there has been a turnover in the Court practice in the USA, where the Supreme Court of Alabama, in the case *Skelton v. Druid City Hospital Board,* had his view for the first time in the Court practice in the USA that a hospital can be characterised as a seller, and stated that in the essence, hospitals are traders. In this Court dispute, the patient sued hospital for the broken needle and it stayed in the patient’s body during his hernia operation. The Court emphasized: we cannot ignore the fact that hospitals are, regardless of being profitable or not, companies. They are not only buildings that offer for placement for seriously ill patients and independent doctors. During their competition, hospitals are presented as public institutions that own knowledge in providing services to patients. The consistent element of this presentation is a guarantee to sell, deliver or provide patients with goods used for providing services and they are appropriate for the intended purpose. In this context, a hospital is clearly “a trader”- in the sense this word has a meaning in a business codex (5).

With the increase of applying technological inventions in the area of health services, there is more transformation in the nature of a doctor’s profession and it used to consist of exclusively offering of health services, to a profession that means a transfer of a huge number of medical means. This statement derives from the fact that hospitals are the only channel through whom a patient can be determined certain, the most often, implantable and prosthetic medical means. If a patient is necessary to have a pacemaker, this implant is the essence of “a transaction” and a patient, not the hospital, who is a final user of this medical means. The essence is not that hospitals are the link in the distribution chain of medical means, but they are, most often, the only possible link in distribution of medical means. Hospitals are not charitable institutions anymore with few doctors and even fewer patients, they are powerful economic subjects today that earn enormous profit from medical “transactions”.
